# Towards Control of a Transhumeral Prosthesis with EEG Signals

**DOI:** 10.3390/bioengineering5020026

**Published:** 2018-03-22

**Authors:** D.S.V. Bandara, Jumpei Arata, Kazuo Kiguchi

**Affiliations:** System Engineering Laboratory, Department of Mechanical Engineering, Kyushu University, Fukuoka 819-0395, Japan; jumpei@mech.kyushu-u.ac.jp (J.A.); kiguchi@mech.kyushu-u.ac.jp (K.K.)

**Keywords:** electroencephalography, motion intention, transhumeral prosthesis, wearable robot, brain computer interface

## Abstract

Robotic prostheses are expected to allow amputees greater freedom and mobility. However, available options to control transhumeral prostheses are reduced with increasing amputation level. In addition, for electromyography-based control of prostheses, the residual muscles alone cannot generate sufficiently different signals for accurate distal arm function. Thus, controlling a multi-degree of freedom (DoF) transhumeral prosthesis is challenging with currently available techniques. In this paper, an electroencephalogram (EEG)-based hierarchical two-stage approach is proposed to achieve multi-DoF control of a transhumeral prosthesis. In the proposed method, the motion intention for arm reaching or hand lifting is identified using classifiers trained with motion-related EEG features. For this purpose, neural network and *k*-nearest neighbor classifiers are used. Then, elbow motion and hand endpoint motion is estimated using a different set of neural-network-based classifiers, which are trained with motion information recorded using healthy subjects. The predictions from the classifiers are compared with residual limb motion to generate a final prediction of motion intention. This can then be used to realize multi-DoF control of a prosthesis. The experimental results show the feasibility of the proposed method for multi-DoF control of a transhumeral prosthesis. This proof of concept study was performed with healthy subjects.

## 1. Introduction

Transhumeral prostheses are worn by upper elbow amputees to substitute for the loss of functions of the upper limb in performing activities of daily living. Early transhumeral prostheses were body powered and capable of providing elbow flexion/extension, wrist flexion/extension, and hand grasping, using a cable operated by glenohumeral articulation of the shoulder [1]. According to the motion intention, the user had to lock joints that were not to be operated. Robotic prosthetic devices have been developed to replace these, and to enable more mobility for their users. Recently, several robotic transhumeral prostheses have been developed [2,3,4,5,6,7,8,9]. These devices generate multi-degree of freedom (DoF) motion and require identification of the motion intention of the user to properly assist the user.

Many of these prostheses are controlled based on surface electromyogram (EMG) signals from residual muscle sites. An EMG signal is a measureable electric current from a muscle capable of providing control signals according to the user’s motion intention [1]. Recently, several studies [2,3,4,10,11,12,13,14,15,16] have investigated EMG-based motion intention estimation techniques for prosthesis control. In [2], in order to control a transhumeral prosthesis, forearm and wrist motions were estimated using an artificial neural network (NN) based on shoulder and elbow motions, and hand motion was generated according to fuzzy rules. In [3], the DEKA arm was proposed with three modular configurations for transradial, transhumeral, and shoulder disarticulated amputees. Here an EMG controller was used in combination with foot controllers and pneumatic bladders for controlling. Lenzi et al. [4] proposed a 5-DoF transhumeral prosthesis for elbow, forearm, wrist, and grasping motions that used an EMG-based low-level controller. In [16], an NN-based method was proposed to estimate distal arm joint angles to control a transhumeral prosthesis using EMG and shoulder orientation. In addition, some studies [11,12,13] report EMG-based motion intention studies for much lower level amputation such as transradial or wrist disarticulation. Despite these advances, there is still a gap to be filled in controlling simultaneous movements in multi-DoF transhumeral prostheses. This is made more challenging because as the level of amputation increases, the number of functions to be replaced by the prosthesis increases, yet fewer muscle sites are available to be used for their control. Further, remaining muscle sites for the prosthetic control are not physiologically related to the distal arm functions [1].

A method to control a multi-DoF transhumeral prosthetic arm has been proposed based on targeted muscle reinnervation [17]. In this method, the residual nerves of the lost muscles are surgically connected to the residual muscles. This allows amputees to contract the reinnervated muscle by attempting to move the missing limb. EMG signals from these muscles can then be used to control prostheses. However, this method is invasive and some difficulties, related to separating the surface EMG signals from different muscles, remain [1]. Owing to the deficiencies in existing methods, electroencephalogram (EEG) is becoming popular among researchers [18,19] for identifying human motion intention for prosthesis control. EEG records electrical signals from the surface of the human skull that carry information related to all bodily motions. In [18], an EEG-based motion estimation method was proposed to control forearm supination/pronation of an artificial arm. Bright et al. [19] proposed a method to control flexion/extension of a prosthetic finger based on EEG signals. Despite these studies, control methods based on EEG for upper limb prostheses lack the capability to control simultaneous multi-DoF motion according to the exact motion intention of the user.

In the present paper, we propose a new hierarchical approach to control a multi-DoF transhumeral prosthesis using EEG signals in combination with residual upper limb motion. The proposed approach comprises three main steps: EEG-based motion intention identification, collection of motion information from healthy subjects to create a database, and estimation of the motion of a prosthesis based on residual limb motion. For a transhumeral amputee, with the available residual limb it is impossible to physically differentiate between the motion intention for a hand reaching motion and that for arm lifting. In a healthy human, hand reaching involves multi-DoF motion of the upper limb, including shoulder, elbow, forearm, and wrist motions. For arm lifting, only the shoulder motion will be involved. Amputees are able to perform only shoulder motions for both actions. Therefore, in the proposed approach, EEG signals are used to differentiate between hand reaching and the arm lifting motion intentions. For this purpose, the effectiveness of two different types of classifiers are compared to learn the dynamic EEG signals related to selected motions. Accordingly, neural networks and *k*-nearest neighbor classifiers are used for motion intention identification. Four different kinds of motion-related EEG features (movement-related cortical potential (MRCP)-based amplitude, delta band power, alpha band power, and root mean square) in time series are provided as inputs to the classifier. The output from the classifier is used in combination with residual limb motion information to estimate elbow motion and hand trajectory, using two different NN-based classifiers. To train these classifiers, motion information collected from healthy subjects is used.

Using the predicted elbow joint angle and the hand trajectory, it is possible to achieve multi-DoF control of transhumeral prostheses for hand reaching or arm lifting. The next section of the paper introduces the proposed methodology for motion intention identification. Section 3 presents the results of the proposed motion prediction method and the motion analysis. This is followed by discussion in Section 4 and conclusions in Section 5.

## 2. Materials and Methods

**Figure 1:**
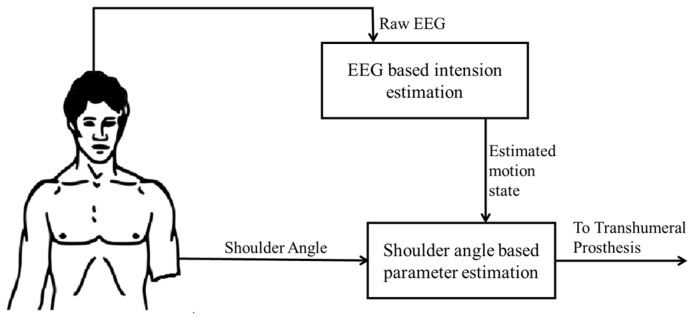
Proposed Hierarchical Approach.

The proposed hierarchical two-stage approach for motion intention identification is shown in Figure 1. In the initial stage, the user’s motion intention to move is estimated using classifiers (neural networks, i.e., multilayer perceptron networks and *k*-nearest neighbors) trained with the features of EEG signals recorded from the scalp of the user. In the later stage, elbow motion and hand endpoint motion is estimated using a separate set of neural-network-based classifiers, which are trained with motion information recorded using healthy subjects. Details of each stage are explained below.

### 2.1. EEG-Based Motion Intention Identification

**Figure 2:**
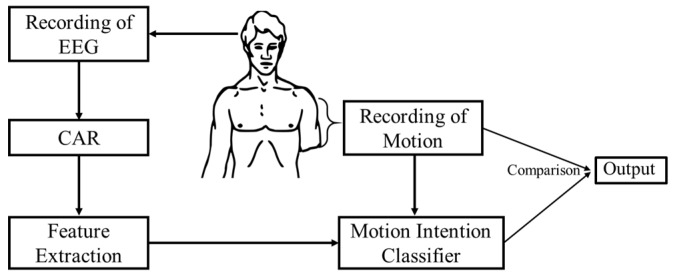
Proposed approach for motion identification. CAR: common average reference.

The main steps and the signal flow chart of the proposed methodology for motion intention identification are shown in Figure 2. Initially, brain activations for the desired motions are recorded experimentally, together with the motion data from the participants. Then the data are preprocessed for feature extraction by averaging. Next, extracted features are used to train the motion intention classifier. Finally, the output from the motion intention classifier is compared with the motion state of the residual limb and the final decision is generated. In this study the effectiveness of an NN-based classifier and a *k*-nearest neighbor classifier are evaluated for the motion intention classifier.

### 2.2. Experimental Setup

**Figure 3:**
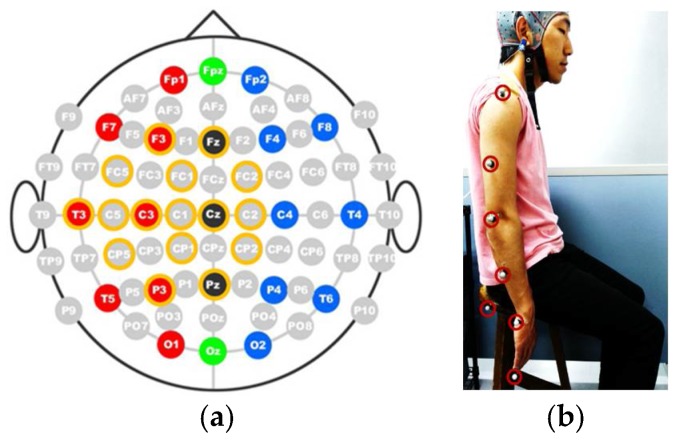
Experimental setup. (**a**) Electrode layout for experiment; (**b**) Marker setup for motion recording.

In the present study, EEG signals were recorded from healthy subjects (4 male, 1 female, age 24–28). A Gamma.cap (Gtec Co., HongKong, China) with 16 electrode locations, a g.Gammabox (Gtec Co.), and a biosignal amplifier (Nihon Kohden Co., Tokyo, Japan) were used to record the EEG signals from the subjects. The standard 10–20 system was followed to place the scalp electrodes into the brain cap. Sixteen electrodes were placed at the Fz, F3, FC2, FC1, FC5, C2, Cz, C1, C3, C5, T3, Cp2, Cp1, Cp5, Pz, and P3 positions, as shown in Figure 3a. The sampling frequency was set to 500 Hz. The right earlobe was used as the reference for EEG recordings.

**Figure 4:**
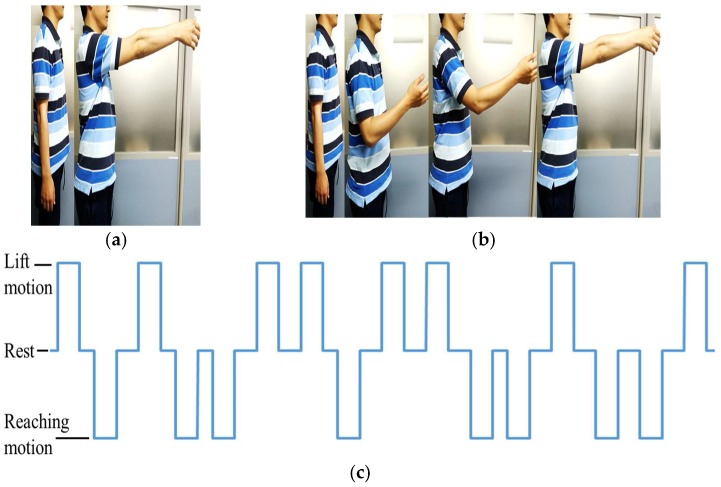
Motions used in the experiment. (**a**) Arm lifting; (**b**) Hand reaching; (**c**) Motion schedule (1 = arm lifting motion, 0 = rest, −1 = hand reaching motion).

To record the motion of the upper limb, a v120: Duo (Optitrack) motion capture system was used. During the experiment, the subject is expected to perform two upper limb motions: arm lifting motion and hand reaching motion. The experiment starts with an audible cue (“start”) and the subject remains seated with arms relaxed at the side for the first 10 s, as shown in Figure 3b. During the arm lifting the subject is instructed to perform only shoulder motions by lifting the arm until it is roughly parallel to the ground (See Figure 4a). The motion is self-phased and afterwards the subject moves the arm back to the resting position. During the hand reaching motion, the subject is instructed to perform a reaching task (similar to a reaching task in activities of daily living, see Figure 4b) until the whole arm is fully extended to make it roughly parallel to the ground. The motion is self-phased and mainly involves shoulder, elbow, forearm, and wrist motions. The subject is instructed to keep his/her eyes closed for the duration of the experiment. During the experiment, an audible cue is given to instruct the subject to perform the arm lifting. To perform the reaching motion, a different audible cue is given. There is a time gap between the two commands, set at random to 5 s or 6 s, to avoid any periodic effects in the EEG signals. The order of the motions is also set at random. During the experiment, 20 motion instances are carried out, 10 for each motion. The motion schedule for the first 16 motions is shown in Figure 4c. The experimental procedure was approved by the Kyushu University ethical review board. All subjects were given detailed written information about the experiments and were given a chance to clarify any doubts. All subjects then signed a consent form to confirm their consent to participation in the experiment.

### 2.3. Data Processing

To minimize the influence of the noise generated among whole electrodes and to normalize the recorded data among every channel, the common average reference (CAR) is calculated with the raw EEG data as follows:

(1) $$e_{car,i}\left(t\right)=e_{i}\left(t\right)-\frac{1}{N}\overset{N}{\underset{k=1}{\sum }}e_{k}\left(t\right)$$

where *N* is the number of channels used in the recordings, $e_{i}\left(t\right)$ is the raw EEG signal from the *i*th channel at time *t*, $e_{car,i}\left(t\right)$ is the CAR-corrected EEG signal of the *i*th channel at time *t*, and $e_{k}\left(t\right)$ is the EEG signal of the *k*th channel for average calculations. After CAR correction, the data are ready for feature extraction.

### 2.4. Feature Extraction

In the present study, four different features are used as inputs to the classifier: movement-related cortical potential (MRCP)-based amplitude, delta band power, alpha band power, and root mean square (RMS).

MRCP can be observed as time domain amplitude fluctuations in the low-frequency delta band and has been used recently [20,21] as a feature which represents motion preparation and execution and contains information related to speed, force, and direction of motions [21]. Therefore, information in the MRCP magnitudes can be used to detect movements or intentions to move. Accordingly, amplitudes of low-frequency delta band signals are used as an MRCP-based feature in this study. CAR-processed EEG signals are passed through a 0.1–2.0 Hz bandpass filter to prepare them for use in classification. Few studies [18,22,23] have used delta band EEG features for motion intention identification. The current study considers the delta band power spectrum and it is obtained by passing the CAR-processed EEG data through a 0.1–4.0 Hz bandpass filter. The resulting signal is squared to obtain the power spectrum for the delta band as explained in [24]. Similarly, alpha band features are used to represent movement intention in some studies [24,25]. For the alpha band power, CAR-processed EEG data are bandpass filtered through 8–12 Hz, followed by squaring. Use of RMS is also reported in a few studies [18]. Accordingly, the effectiveness of RMS will also be evaluated in the study. RMS is calculated as

(2) $$RMS=\sqrt{\frac{1}{N_{a}}\overset{t}{\underset{k=t-N_{a}+a}{\sum }}e_{ik}^{2}}$$

where *e_ik_* is the EEG signal of the *i*th channel after filtering the *k*th sample, and *N_a_* is the sampling number which is selected based on observations for low noise and high activation, from one of three choices: 100 ms, 200 ms, or 400 ms. In the present study, *N_a_* is selected to be 400 ms. In addition, the filtering techniques used for the bandpass filter are based on finite impulse response filters, which in general form are presented as [26]

(3) $$y\left[n\right]=\;\overset{M-1}{\underset{k=0}{\sum }}b_{k}x\left(n-k\right)$$

where as the parameters are computed automatically during the implementation of the filter using the EEGLAB [26] toolbox for a given signal.

**Figure 5:**
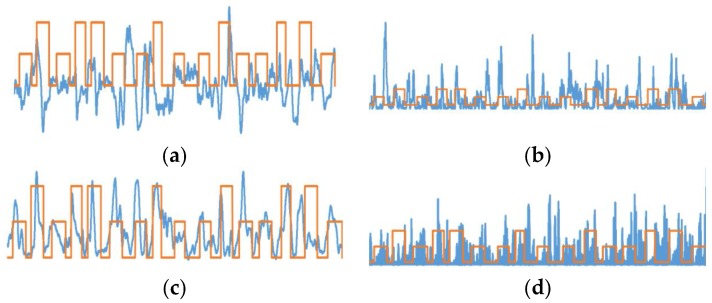
Feature plots for channel selection. (**a**) Movement-related cortical potential (MRCP); (**b**) delta band power; (**c**) RMS; (**d**) alpha band power.

After extracting the features for each subject, the features are plotted for each channel. These plots are observed and two prominently activated channels are selected for classification of the motion. By observation, FC2 and C2 locations were selected for MRCP-based amplitudes, RMS, and delta power band features. With alpha power band, the highest activations were observed for P3 and Pz locations. Sample feature plots for MRCP amplitudes, delta band power, RMS, and alpha band power are shown in Figure 5a–d, respectively.

Using the selected channels, a time-delayed feature matrix is prepared as the input to the classifier as follows for both training and testing phases:

(4) $$Feature\;Matrix=\;\left[\begin{array}{c} EEG_{i}\left(t\right)\\ EEG_{i}\left(t-\Delta t\right)\\ EEG_{i}\left(t-2\Delta t\right) \end{array}\;\begin{array}{c} EEG_{i}\left(t+\Delta t\right)\\ EEG_{i}\left(t\right)\\ EEG_{i}\left(t-\Delta t\right) \end{array}\;\begin{array}{c} EEG_{i}\left(t+2\Delta t\right)\\ EEG_{i}\left(t\right)\\ EEG_{i}\left(t\right) \end{array}\right]$$

where $EEG_{i}\left(t\right)$ is the EEG feature in the selected *i*th channel at time *t.* The time delay Δ*t* is determined by performing classification on three data sets selected at random for different Δ*t* values of 100 ms, 250 ms, 500 ms, or 1000 ms. After the random evaluation, Δ*t* was selected to be 250 ms for the whole study (for both training and testing) since this value results in the highest classification accuracy. Using time-delayed inputs of the same channel to the input matrix will help the classifier to learn the dynamic information contained in the EEG signals.

### 2.5. NN-Based Motion Intention Estimation

Artificial NNs have been widely used to solve different classification problems. NN-based classification includes training and prediction phases. During the training phase, an input feature matrix similar to Equation (4) is fed into a separate feedforward NN for each subject. Each NN consists of three layers: the input layer, a hidden layer, and the output layer. The hidden layer contains 30 neurons. The sigmoidal transfer function is used as an activation function in both the hidden and output layers, to calculate the output of each layer. The output from the NN is the estimated motion intention of the user: arm lifting motion, hand reaching motion, or rest. Each NN is trained using the error backpropagation algorithm with feature matrices as the input. From the recorded data, 80% of the data was used for training and the remaining 20% of the data was used for the testing of the classifiers.

During NN training, a value is assigned to each motion: 1 for arm lifting motion, −1 for hand reaching motion, and 0 for resting, as shown in Figure 2. The output prediction from an NN is also a value from −1 to 1, representing the above classes. For each of the five subjects, four different NNs are trained—one for each feature—for a total of 20 NNs. In addition, for each subject, a different NN was trained using all four feature matrices as inputs. This network contained 80 hidden neurons. Five NNs were trained separately for all five subjects.

### 2.6. *k*-Nearest Neighbor Classifier-Based Motion Intention Estimation

The *k*-nearest neighbor (*k*-nn) algorithm is a widely used simple classification technique that finds the *k*-many nearest neighbors in a training data set and then maps them the same during the estimation process. The *k*-nn algorithm is widely presented in following strategies [27]:

(5) $$y\left(d_{i}\right)=argmax_{k}\sum_{X_{j}\in kNN}y\left(x_{j},c_{k}\right)$$

(6) $$y\left(d_{i}\right)=argmax_{k}\sum_{X_{j}\in kNN}Sim\left(d_{i},x_{j}\right)y\left(x_{j},c_{k}\right)$$

where $d_{i}$ is a test document, $x_{j}$ belongs to class $c_{k}$, and Sim($d_{i},x_{j})$ is the similarity function for $d_{i}$ and $x_{j}$. In the two strategies, as in Equation (5), the prediction class will be the class that has the largest number of members in the *k*-nn and, as in Equation (6), the prediction will be the class with the maximum sum among the *k*-nn. However, it should be noted that the value of *k* is important for better performance in the classification. Therefore, during the implementation phase of the *k*-nn classifier with MATLAB, an optimization process also runs simultaneously with the classifier. The optimization algorithm automatically determines the best *k* value and the best metric to be used for the *k*-nn classifier based on the optimization results, suitable for each training data set of each feature per subject. During the optimization, parameters are optimized to minimize the fivefold cross-validation loss. Training and testing data were prepared in a similar manner to the NN-based classification. Twenty different *k*-nn models were trained for all the five subjects and for the four features used. In addition, five additional *k*-nn models were generated by training a single *k*-nn model for each subject by combining all the four feature matrices. The trained *k*-nn models were used to estimate the motion intention.

### 2.7. Comparison

The proposed approach includes a step to compare residual limb motions with the NN output, to improve the accuracy of the prediction. This prevents false triggers when the user does not want to perform any motion. The final prediction of the proposed method is decided based on the result from the comparison. Based only on the movement of the residual limb, it is impossible to identify desired motions. However, the proposed method is capable of identifying user intention to move the upper limb. Thus, the rules of the comparison are shown in Table 1.

### 2.8. Motion Analysis

**Figure 6:**
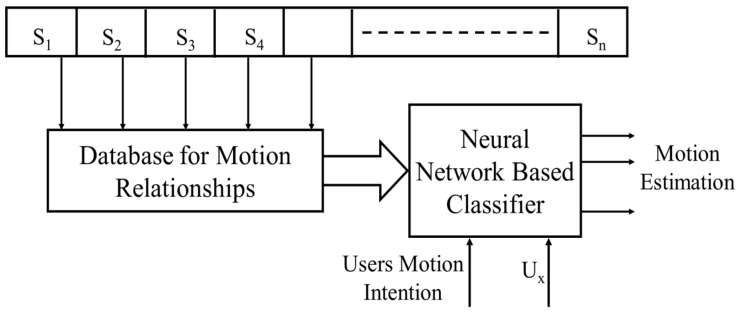
Proposed approach for estimation of identified motion (S_n_—healthy individuals, n—1, 2, 3, …, U_x_—residual limb joint angle of the transhumeral amputee).

In the approach proposed above, it is insufficient to identify only the motion intention of the transhumeral amputee for prosthetic control. Therefore, after identifying the motion intention of a reaching motion, the method shown in Figure 6 is used to estimate the motion of the prosthesis.

A transhumeral amputee with a residual limb is typically only able to make shoulder motions. Therefore, in this method, initially a database is created from the motion information of the desired motions of healthy subjects (S_1_, S_2_, …, S_n_) to identify the relationships between shoulder motions and distal motions of the upper limb. With motion capturing, it is possible to record the motion of the upper limb joints experimentally. This information is used to derive the relationships between shoulder motions and distal motions of the upper limb (i.e., shoulder joint angle (U_x_) with respect to the elbow joint angle and shoulder joint angle (U_x_) with respect to the end point motion of the upper limb). Later, information in the database is used to train a different set of NN-based classifiers to estimate the motion of the prosthesis.

Accordingly, 10 different NNs are trained using the joint relationship information obtained during the motion analysis. Five of them are to estimate the elbow joint angle for each subject, the remaining five are to estimate the hand trajectory for each subject. Each NN is trained with data from four subjects, with one subject excluded in each instance. The excluded subject is assumed to be the amputee; the remaining four subjects are assumed to be healthy subjects. The NNs include three layers: input, hidden, and output layers. Each NN is provided with an input from the estimated motion from the previous classifier (1 = arm lifting motion, 0 = rest, −1 = hand reaching motion) and three inputs of shoulder joint angles positioned at 0 ms, 250 ms and 500 ms. One set of NNs is trained to estimate the elbow joint angle using the error backpropagation algorithm. The other set is trained to estimate the hand trajectory values for the *x* and *y* directions. The hidden layer comprises 10 neurons. Output from the NNs are the elbow joint angle for the prosthesis to be controlled and hand trajectory values. For clarity in this paper, a summary of the NN configurations presented in this study are shown in Table 2.

## 3. Results

### 3.1. NN-Based Motion Intention Estimation

**Figure 7:**
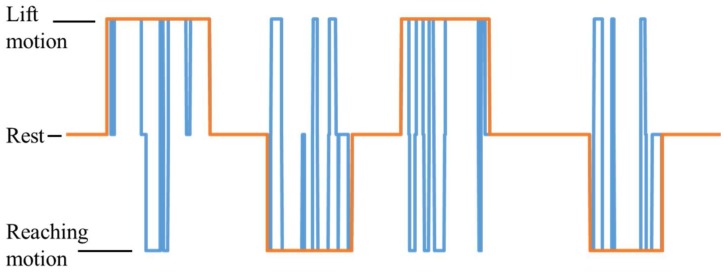
Prediction from the system with MRCP for Subject 1 (1 = lifting motion, −1 = reaching motion, 0 = rest, blue line = prediction, orange line = subject motion).

In total, 20 artificial NNs were trained to predict motion intention for lifting and reaching motions of the upper limb using EEG signals. The outputs from these NNs were then compared with the residual limb motion, as shown in Table 1. The resulting output for Subject 1 for the MRCP is shown in Figure 7. The results for all five subjects are summarized in Table 3. For the delta band power, the highest accuracy of 75.7% was achieved with Subject 2. The average accuracy for the delta power band was 71.1%. When alpha band power is used, the highest accuracy of 78.9% was recorded from Subject 3. The average was recorded as 73.4%. With the MRCP-based feature, the highest accuracy of 84.7% was achieved with Subject 1, and the recorded average was 73.4%. A highest accuracy of 82.0% was achieved for Subject 1 with RMS as the feature. The average for RMS was 63.7%. When all features are combined for training, the highest accuracy of 80.9% was recorded with Subject 1 and an average accuracy of 72.5% was recorded.

### 3.2. k-NN-Based Motion Intention Estimation

Similar to NN-based classifiers, 20 *k*-nn classifiers were trained to predict the motion intention for lifting and reaching motions. During the prediction, the outputs from the *k*-nn models were compared with the residual limb motion, as in Table 1. Summarized results for the *k*-nn-based motion estimations are shown in Table 4. When the delta power band was used to estimate the motion intention, the highest accuracy recorded was 63.6% for Subject 1 with an average of 58.7% for the five subjects. With the alpha power band, Subject 1 recorded a highest accuracy of 59.7% and the recorded average accuracy was 54.8%. The MRCP-based feature resulted in a highest accuracy of 70.0% for Subject 1 and an average accuracy of 60.9% for all five subjects. RMS recoded a highest accuracy of 72.6% for Subject 1. The recorded average was 61.3% for all the five subjects. In addition, when all the features were combined to estimate the motion intention, a higher accuracy of 65.8% was recorded from Subject 1. The recorded average was 59.6%.

### 3.3. Motion Relationships

**Figure 8:**
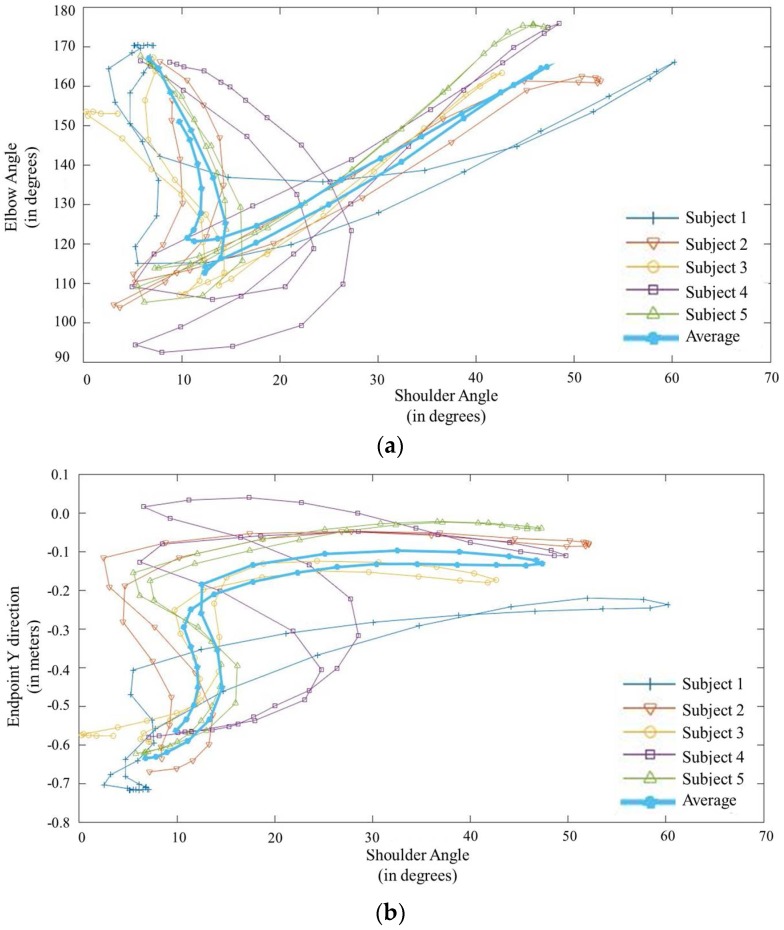
Motion relationships. (**a**) Elbow flexion/extension angle to the shoulder flexion/extension angle for reaching. (**b**) Variation of the endpoint with the shoulder flexion/extension angle.

It is important to identify the relationship between the shoulder angle and the elbow angle for prosthetic control. The measured relationship is shown in Figure 8a. The figure shows the variation of the elbow flexion/extension angle with respect to the shoulder flexion/extension angle of the residual limb for each subject. It also shows the average variation for all subjects. For the lifting motion, the elbow angle remains constant at the fully extended position. However, for the reaching motion, the angle initially decreases rapidly and then increases until the elbow is fully extended. It is also important to realize the relationship between shoulder flexion/extension angle and the desired end effector position for prosthesis control. Figure 8b shows the variation of the endpoint of each subject with the shoulder joint angle. It also shows the average for all subjects. For the reaching motion, the end effector position of the hand reaches its maximum point during the initial 20 degrees of shoulder motion. Throughout the rest of the shoulder motion, the endpoint remains at the same point. In the reverse motion, the endpoint remains at it maximum point until the shoulder reaches 20 degrees and it returns to the starting point within the final 20 degrees of shoulder motion.

### 3.4. Motion Estimation

**Figure 9:**
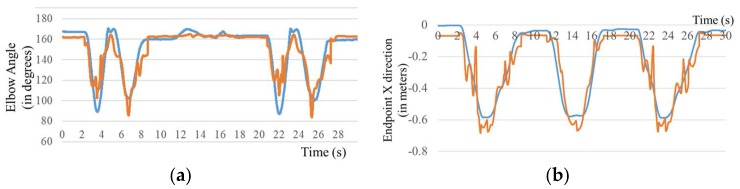

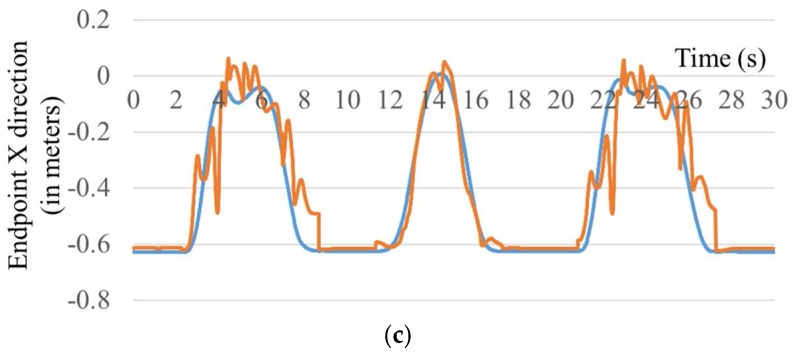
Comparison of data measured experimentally (blue line) and those generated by the neural network classifier (orange line). (**a**) Estimated elbow flexion/extension angle; Hand trajectory in the (**b**) *x* direction and (**c**) *y* direction.

NN-based classifiers trained with the motion relationships of healthy subjects were used to estimate the elbow flexion/extension and hand trajectory of the transhumeral prosthesis. The generated results are compared with the motion-captured experimental results. Similarly, generated elbow flexion/extension results for Subject 1 are shown in Figure 9a. The joint angle generated by the classifier is similar to that generated experimentally. Similar results were obtained for the other four subjects. Figure 9b,c shows that the hand trajectory generated by the classifier is similar to that generated experimentally. For the remaining four subjects, similar results were obtained.

## 4. Discussion

**Figure 10:**
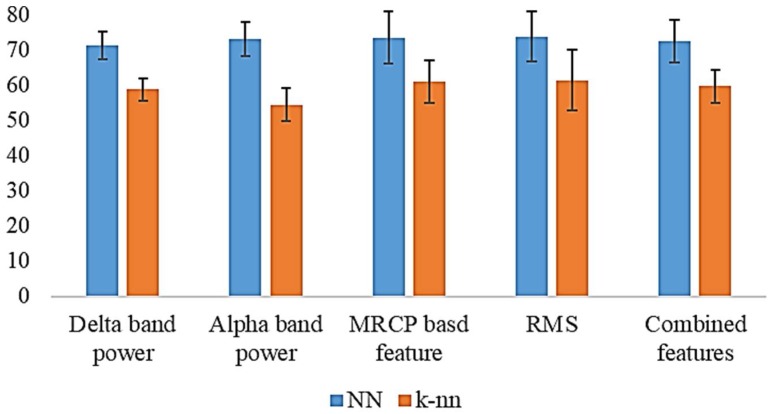
Summary of the results of the motion intention classifiers (error bars show the standard deviation).

In this study, a hierarchical two-stage motion prediction approach was proposed for controlling a transhumeral prosthesis. Initially, the user’s motion intention for reaching or lifting of the arm was identified using the EEG recording from the scalp. For this purpose, four different kinds of EEG features were used to train two different classification techniques. Identified locations for feature extraction were related to motor cortex areas of the brain, which will be activated for motor tasks. Accordingly, as the motion intention classifier, 20 different NN models and 20 *k*-nn models were trained with features of MRCP-based amplitudes, delta band power, RMS, and alpha band for the five subjects. Trained classifiers were used to predict three different motion classes: hand reaching, arm lifting, and rest. To improve the reliability of the estimation, the output from these classifiers was compared (gated) with motions of the residual limb. A summary of the results is shown in Figure 10. NN-based motion estimation performed much better than the *k*-nn-based motion estimation. With the NN-based estimation, the MRCP-based feature, RMS, and alpha band power showed almost equal average results for all the five subjects. However, alpha band power had the least deviation in the results. A highest accuracy was achieved with the MRCP-based feature for Subject 1 (84.75%); the lowest was with RMS for Subject 5 (61.6%). Conversely, with *k*-nn-based estimation, RMS achieved the highest average accuracy (61.3%) with a higher deviation. When features were combined, the average accuracy was lower than for the other features, except for the delta band power with NN-based motion estimation. With *k*-nn-based motion estimation, models trained with combined features recorded average accuracies higher than those of alpha band power and the delta band power. In this study, analysis was carried out by combining all four feature types. However, there are a number of different possibilities for combining the features, such as combining two or three different features. In the scope of this paper, we do not discuss these possibilities.

In addition, the chance levels were computed for the five subjects; they have the values of 46.9%, 49.0%, 53.4%, 36.9%, and 37.5% from Subjects 1–5, respectively. Percentage accuracy values recorded with the neural-network-based estimation are significantly higher than the recorded chance levels for all five subjects. Furthermore, a *p*-value < 0.05 recorded based on the binomial test suggests that the results obtained are statistically significant.

The relationships among residual shoulder angle, elbow joint angle, and end effector position were also investigated. These relationships were used to estimate the end effector position and the elbow joint angle of the amputee using the residual shoulder angle. Ten individual NNs were trained with healthy subjects to estimate the end effector position and the elbow joint angle. These results show that it is feasible to control the elbow joint of a transhumeral prosthesis, once the motion intention is identified. It is also possible to control multi-DoF motion of the prosthesis for reaching tasks. However, this requires a properly developed inverse kinematic model for the prosthesis, using the relationship between the endpoint and the residual shoulder angle. Thus, the proposed method demonstrates the capability of using the proposed approach to control multiple DoFs of a transhumeral prosthesis.

However, this proof of concept study was performed with healthy subjects. In [28], Estelle et al. showed that amputees show deteriorated activations of the EEG signals compared with healthy subjects during motor execution tasks of absent movements of the individual joints of the phantom limb. In the current study, during arm lifting and reaching movements, both phantom arm movement and the residual limb movements are collaborated. It is not clearly understood what will be the response of the brain in such a scenario. On the other hand, some studies [29,30] have shown that the involvement of the brain to perform task-based upper limb motions such as reaching, pointing, etc., is different from the individual joint motions. On this note, we assume that the current study is applicable to upper limb amputees.

## 5. Conclusions

In this paper, a new approach was proposed to control a multi-DoF transhumeral prosthesis taking into account the motion intention of the user based on EEG signals. It consists of three major steps: EEG-based motion intention identification, collection of motion information from healthy subjects to create a database, and estimation of the motion of the prosthesis based on residual limb motion. The motion intention was predicted for two major upper limb functions: arm lifting and hand reaching. Based on the motion intention prediction and the residual limb shoulder angle, appropriate multi-DoF motion of the arm prosthesis can be realized. To predict the motion intention, four different features were used to provide input to the NN-based and *k*-nn-based classifiers. Time-delayed inputs were provided to the classifier to yield dynamic information of the different features. The prediction from the classifier was compared with the residual limb motion to generate a final prediction of motion intention. The results prove the feasibility of the proposed approach to control multi-DoF motion of a transhumeral prosthesis using EEG signals.

## Figures and Tables

**Table 1:** Rules of comparison for residual limb motion and the neural network (NN) output.

Output from NN	User’s Desire for Motion	Final Prediction
Rest	no	Rest
Hand reaching motion	no	Rest
Arm lifting motion	no	Rest
Rest	yes	Rest
Hand reaching motion	yes	Hand reaching motion
Arm lifting motion	yes	Arm lifting motion

**Table 2:** Summary of configuration of proposed Neural Networks (NN).

	Neural Network for Motion Intention Prediction	Neural Network for Motion Estimation of Prosthesis
Number of NNs	25 (5 per feature set for 4 features and 5 NNs for combined feature matrix)	10 (5 for elbow angle, 5 for end point estimation)
Input to NN	Feature matrices of EEG features of MRCP, Delta Power, and RMS	Shoulder Angle of the Healthy Subject from the collected database
Hidden Layers motion	30	10
Output	User‘s motion intention for reaching or lifting motions	Elbow angle and end point position

**Table 3:** Summarized results for five subjects with neural networks (accuracy percentage).

	Delta Band Power	Alpha Band Power	MRCP-Based Feature	RMS	Combined Feature
Subject 1	72.4	78.1	84.7	79.4	80.9
Subject 2	75.7	72.4	71.1	82.0	71.6
Subject 3	74.5	78.9	78.7	71.0	75.9
Subject 4	66.8	66.1	68.6	74.4	71.3
Subject 5	66.1	70.3	63.9	61.6	62.7
Average	71.1 ± 3.9	73.1 ± 4.8	73.4 ± 7.4	73.7 ± 7.2	72.5 ± 6.0

**Table 4:** Summarized results for five subjects *k*-nn (accuracy percentage).

	Delta Band Power	Alpha Band Power	MRCP-Based Feature	RMS	Combined Feature
Subject 1	63.6	59.7	70.0	72.6	65.8
Subject 2	58.9	53.0	62.8	67.9	55.8
Subject 3	59.6	55.0	62.0	61.6	61.0
Subject 4	57.4	46.5	58.8	56.6	62.6
Subject 5	53.9	57.9	51.0	47.9	52.7
Average	58.7 ± 3.2	54.4 ± 4.6	60.9 ± 6.2	61.3 ± 8.6	59.6 ± 4.7
